# Retrospective case series of high-density silicone oil (Oxane HD) in severe proliferative vitreorretinal retinal detachment patients

**DOI:** 10.1186/s40942-024-00548-2

**Published:** 2024-04-11

**Authors:** Ramon Antunes De Oliveira, Vinicius Oliveira Pesquero, Lucas Zago Ribeiro, Murilo Ubukata Polizelli, Aalec Rinhel Souza Ferreira Da Silva, Nilva Simeren Bueno De Moraes, Rodrigo Antonio Brant Fernandes, Octaviano Magalhaes Junior, Mauricio Maia

**Affiliations:** 1 Department of Ophthalmology, Universidade Federal de São Paulo (UNIFESP/EPM), São Paulo, Brazil; 2 Universidade Federal do Triângulo Mineiro (UFTM/FMTM), Minas Gerais, Olhos Oeste Paulista, Assis, Brazil; 3 Hospital de Olhos Oeste Paulista, Assis, Brazil

**Keywords:** Heavy silicone oil, Proliferative vitreoretinopathy, Rhegmatogenous retinal detachment

## Abstract

**Background:**

Describe complications and clinical outcomes of heavy silicone oil (HSO) Oxane HD® use as an alternative to overcome the challenges of performing vitrectomy to treat tractional and rhegmatogenous retinal detachments with proliferative vitreoretinopathy (PVR).

**Methods:**

A retrospective, observational study was performed on patients from one center from August 2014 to Aug 2023. It was included patients who underwent surgery using HSO Oxane HD® to treat rhegmatogenous retinal detachment with PVR or mixed tractional and rhegmatogenous diabetic retinal detachment. Severely ill patients who could not attend to follow up were excluded. The primary outcome was successful retinal attachment at first postoperative month. A descriptive analysis was performed.

**Results:**

Among the 31 patients, 29 (93.5%) underwent surgeries due to rhegmatogenous retinal detachment and two (6.5%) for diabetic retinal detachment. The primary anatomic success was achieved in 27 (87.1%) patients. At the final visit, 17 (56.6%) had vision better than 20/400 (range, 20/30 to light perception). The vision was stable or improved in 22 (76.8%) patients at the end of follow-up. Nineteen (61.3%) patients required hypotensive eye drops after HSO use and twelve (38.7%) still required hypotensive eye drops at the final follow-up; three (9.7%) patients required additional glaucoma surgeries.

**Conclusions:**

HSO is safe and useful for complex retinal detachments cases specially with inferior tears and PVR. Ocular hypertension is frequent and usually clinically controlled with hypotensive eyedrops. Close postoperatively follow-up is advised due to the ocular complications, particularly elevated intraocular pressure and emulsification.

Vitrectomy, the most common surgical technique performed to treat rhegmatogenous retinal detachment, can be combined with phacoemulsification and/or scleral buckling in selective cases to optimize surgical results. Surgical skill and the choice of proper vitreous substitutes are crucial for the final visual outcomes. The most common vitreous substitutes used in vitreoretinal surgeries are air, expandable gases, and silicone oil (SO).

SO, which is hydrophobic and lighter than water, tends to float in the vitreous cavity and provides a good tamponade effect in the superior retina. However, it leaves an inferior residual meniscus that can accumulate fluid, proinflammatory proteins, and cellular elements such as retinal pigment epithelial tissue [1, 2]. This facilitates proliferative vitreoretinopathy (PVR) formation and recurrent retinal detachments which are the main reasons for surgical failure and blindness [3–5].

Heavy SO (HSO), which has been described since the late 1990s, is characterized by a density that exceeds 1, the ability to tamponade the inferior retina in horizontal gaze, and prevention of retinal reproliferation and redetachment [6]. It is advocated for retinal detachments with large inferior breaks or inferior PVR in patients unable of proper head positioning, such as spinal osteoarthritis, or cognitive impairment [1].

The current study is a nine year retrospective case series of complicated retinal detachments using HSO and their clinical outcomes.

## Methods

We retrospectively reviewed 31 patients from August 2014 to Aug 2023 (9 years) from one center in Assis, Sao Paulo, Brazil. The primary outcome was primary retinal attachment at first postoperative month. The inclusion criteria were rhegmatogenous retinal detachment with proliferative vitreoretinal disease or mixed tractional and rhegmatogenous diabetic retinal detachment treated with HSO (Oxane HD®, Bausch & Lomb, Toulouse, France). The patients had poor clinical prognoses and were informed of probable global atrophy due to anterior PVR and ciliary body traction resulting from advanced PVR. Severely ill patients who could not attend regular follow-up examinations were excluded.

**Fig. 1:**
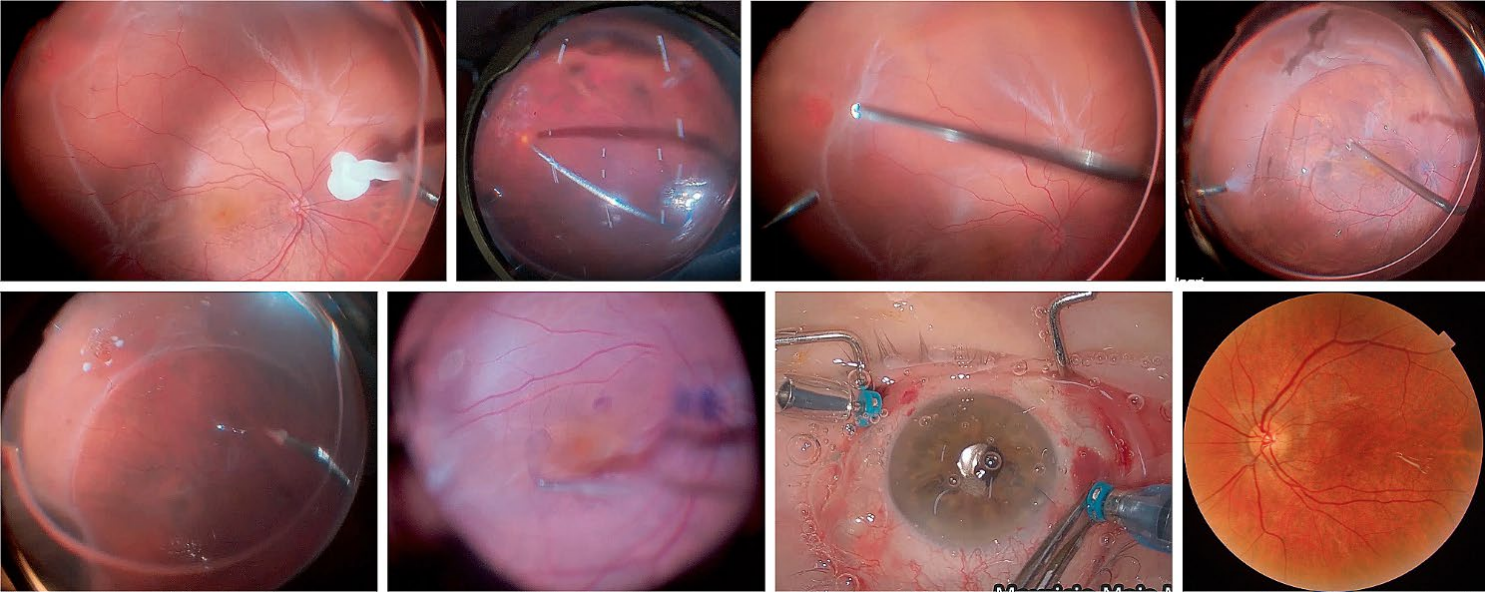
Fundus photography with retinal detachment (**A**), after surgery with HSO (**B**)

One surgeon (MM) performed all four-port pars plana vitrectomies with Constellation® Vision System (Alcon, Fort Worth, Texas, USA) and a chandelier illumination. The surgery was combined with phacoemulsification and/or scleral buckling at the discretion of the surgeon. In all cases which perfluorocarbon liquids (PFCL) were used, they were aspirated during fluid-air exchange and then the Oxane HD® HSO was injected (example in Fig. 1). The HSO was removed at the surgeon’s discretion based on the retinal status, intraocular pressure (IOP), and visual prognosis after several weeks in another surgical procedure.

The HSO removal was performed on bimanual technique and under direct visualization. A 18-gauge needle was connected to the extrusion line through one sclerotomy and another 18-gauge needle was connected to the vitreous cutter aspiration line. This technique is important for fast and safe HSO removal because extrusion line frequently partially or completely clogs during HSO aspiration, and the other needle keeps a continuous aspiration inside the SO bubble during the whole procedure. This prevents the release of a remaining HSO bubble back to the posterior pole [7].

Patients were evaluated on the postoperative day 1, week 1, months 1 and 3, and every trimester thereafter. Additional visits were scheduled as needed according to surgical complications. Best corrected visual acuity (BCVA), anterior biomicroscopy, IOP, and fundus photographs were performed at all appointments and further ancillary examinations in specific cases.

The preoperative and postoperative BCVA were analyzed after conversion to the logarithm of minimum angle of resolution (logMAR) BCVA. The results were detailed in a descriptive analysis.

The local ethics committee approved the study, which followed the tenets of the Declaration of Helsinki.

## Results

Data from 31 patients (mean age, 58.8 ± 13.0 years; 21 men, 67.7%) were analyzed (Table 1). Of the 31 patients, 29 (93.5%) needed surgery to treat a rhegmatogenous retinal detachment and two (6.5%) for a tractional retinal detachment with previously glaucoma drainage device for neovascular glaucoma secondary to diabetic retinopathy.

**Table 1:** Characteristics of patients and pre and postoperative’s eye status

n	31
Age (years) (mean ± SD)	58.8 ± 13.0
Gender (male)	21 (67.7%)
Rhegmatogenous retinal detachment	29 (93.5%)
Diabetic retinal detachment	2 (6.5%)
Right eye	17 (54.8%)
Phakic at first surgery	17 (54.8%)
Pseudophakic at first surgery	12 (38.7%)
Aphakic at first surgery	2 (6.4%)
Follow up (months)	27.3 ± 23.4
**Number of previous retinal surgeries**	
0	15 (48.4%)
1	10 (32.2%)
2	5 (16.1%)
3	1 (3.4%)
Hypothensive eye drops after Oxane	19 (61.3%)
Hypothensive eye drops last visit	12 (38.7%)
Glaucoma Surgery	3 (9.7%)

Seventeen (54.8%) surgeries were performed in the right eye. Seventeen eyes (54.8%) were phakic and underwent phacoemulsification during the first surgery; Twelve (38.7%) were pseudo phakic, and two (6.4%) were aphakic. Seven (22.6%) patients underwent scleral buckling. The mean follow-up was 27.3 ± 23.4 months (range, 2-101 months) after HSO infusion.

No previous retinal surgery had been performed in fifteen (48.4%) patients; ten (32.2%) had undergone one retinal surgery, five (16.1%) had two previously retinal surgeries, and one patient (3.2%) had three previously retinal surgery before the HSO injection (Table 1).

**Fig. 2:**
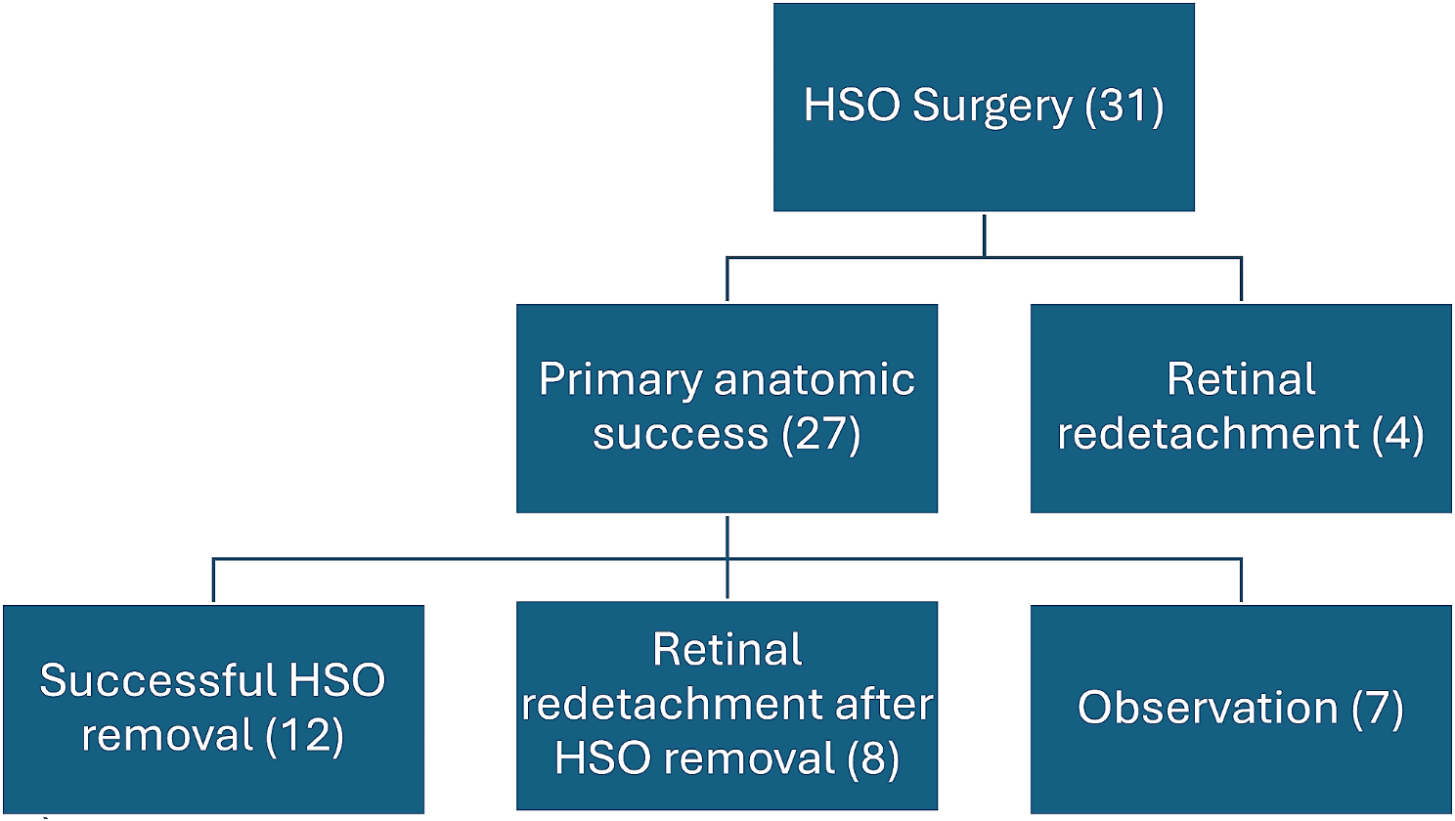
Flow chart of the anatomical outcome after HSO use in complex retinal detachments cases

Twenty-seven (87.1%) patients had primary anatomic success at the one-month postoperative clinical examination. Patients were scheduled for HSO removal depending on surgeon and patients’ agreement regarding retina status, visual prognosis and patient’s health and social availability: eleven patients had their HSO removed before 3 months, four patients between 4 and 6 months and nine patients more than 6 months after first surgery, seven patients did not remove HSO. The outcomes of these patients were: twelve kept their retina attached after HSO removal; eight had retinal redetachment after HSO removal and were retreated with standard SO or HSO; four patients had early retinal redetachment and were retreated with standard or HSO (primary anatomic failure), seven patients had not been submitted to further surgery (five patients refused HSO removal due to bad visual prognosis and were kept under close surveillance and two patients had short follow up period − 2 and 5 months (Fig. 2).

The mean baseline BCVA was 1.93 ± 1.17 logMAR and ranged from 20/30 to light perception. Ten (37%) patients had a BCVA better than 20/400 at baseline. At the final visit, the mean BCVA was 1.65 ± 1.06 logMAR. Fifteen (50%) patients had a BCVA better than 20/400 (range, 20/30 to light perception) at final visit.

After HSO surgery, 19 (61.3%) patients needed hypotensive drops for IOP control. The IOP rise was clinically well controlled and was not the reason for HSO removal in any patient. At the final follow-up, a total of 12 (38.7%) patients still required hypotensive eye drops and three (9.7%) patients had required additional glaucoma procedures: one patient needed selective laser trabeculoplasty, and two patients needed cyclophotocoagulation. These three glaucoma procedures were performed several months after HSO removal or HSO/SO exchange and all these patients had previously silicone oil emulsification. Two (6.4%) patients had been previously submitted to glaucoma drainage device implant surgery in the superior quadrant before the tractional retinal detachment. Other postoperative complications: one (3.2%) patient developed severe posterior capsule opacification; six (19,3%) patients developed clinically visible SO emulsification; two (6.4%) patients developed epiretinal membrane, two (6.4%) patients developed corneal decompensation, one (3.2%) patient had chronic hypotony and globe atrophy; two (6.4%) patients developed optic nerve atrophy (Table 2).

**Table 2:** Pre-and postoperative records per patient

Indication	Age/ gender	Lens Status	Time to HSO (months)	IOP after Oxane	Number IOP Meds	IOP Final	Number IOP Meds	Time Follow up (months)	Baseline VA	Final VA	Comments
RRD	60/ M	Phakic	7	11	0	17	0	42	20/200	20/60	ERM
RRD	75/ F	Pseudo	6	16	1	16	0	10	CF 1 M	20/160	
RRD	55/ M	Phakic	15	16	2	18	2	18	CF 1 M	CF 2 M	
RRD	64/ F	Phakic	2	14	0	16	0	9	HM	ND	
RRD	55/ M	Pseudo	10	13	0	13	0	4	HM	HM	
RRD	50/ M	Phakic	6	13	0	13	0	7	HM	20/100	
RRD	38/ M	Phakic	13	10	0	16	0	61	20/150	20/160	
RRD	24/ F	Phakic	5	14	2	23	1	8	HM	20/400	
RRD	58/ F	Phakic	2	10	4	21	2	3	HM	20/200	
RRD	67/ M	Phakic	9	20	2	24	2	36	CF 0.1 M	CF 1 M	SO emulsification, SLT after DEX implants, after SOR
RRD	57/ M	Phakic	3	20	3	14	0	12	ND	20/50	RRD secondary to uveitis
RRD	57/ M	Aphakic	3	14	2	0	0	5	ND	LP	Aphakic, globe atrophy
RRD	65/ M	Pseudo	NA	9	1	13	0	25	20/60	20/80	Permanent Oxane
RRD	50/ F	Pseudo	NA	12	0	10	0	39	CF 1 M	HM	Previous IONN, severe PCO, Permanent SO
RRD	53/ M	Phakic	NA	12	0	12	0	2	HM	20/400	Lost follow up
RRD	45/ M	Aphakic	NA	16	3	14	2	38	HM	LP	Bullous keratopathy, Permanent Oxane
RRD	52/ F	Phakic	NA	14	0	12	0	24	HM	HM	Permanent Oxane
RRD	77/ M	Pseudo	NA	12	2	13	0	5	ND	20/120	
RRD	88/ F	Phakic	1	14	4	13	2	52	HM	HM	AMD macular scar, Oxane removed, Permanent SO
RRD	76/ M	Pseudo	4	ND	ND	14	0	4	ND	CF 2 M	Previous corneal graft and advanced AMD
RRD	40/ F	Phakic	11	12	2	16	0	60	20/30	20/160	SO emulsification, Ciclophotocoagulation
RRD	68/ F	Pseudo	6	13	3	12	2	10	20/100	20/30	
RRD	58/ M	Phakic	13	16	3	17	3	25	20/125	20/100	
RRD	70/ M	Pseudo	18	18	0	11	0	39	20/100	CF 1 M	Oxane removed, Permanent SO
RRD	47/ M	Pseudo	3	16	1	13	1	13	20/30	MM	Oxane removed, Permanent SO
RRD	64/ F	Phakic	2	15	1	15	1	25	CF 0.5 M	CF 1 M	SO emulsification, Oxane removed, Permanent SO
RRD	66/ M	Pseudo	15	20	0	20	0	101	20/40	LP	SO emulsification, IONN, Oxane removed, Permanent SO
RRD	66/ M	Pseudo	9	14	0	12	0	8	HM	CF 1 M	ERM
RRD	47/ M	Pseudo	7	10	1	17	0	21	20/50	20/80	
TRD	66/ M	Phakic	NA	14	2	13	2	55	LP	LP	Previous GDD for DR NVG, SO emulsification, Permanent Oxane
TRD	66/ M	Phakic	34	20	2	14	2	63	HM	20/400	Previous GDD for DR NVG, SO emulsification, Permanent SO, Ciclophotocoagulation, IONN, Corneal graft

HSO: heavy silicone oil; IOP: intraocular pressure; Meds: medications; VA: visual acuity; CF: count fingers; HM: hand motion; LP: light perception; RRD: rhegmatogenous retinal detachment; TRD: tractional retinal detachment; M: male; F: female; Pseudo: pseudophakic; NA: not applicable; ND: No data; ERM: epiretinal membrane; SO: silicone oil; SLT: selective laser trabeculoplasty; DEX: dexamethasone; SOR: silicone oil removal; IONN: ischemic optic nerve neuropathy; PCO: posterior capsule opacification; AMD: age macular degeneration; GDD: glaucoma drainage device; DR: diabetic retinopathy, NVG: neovascular glaucoma

## Discussion

The background for the use of HSO is their ability to concentrate inferiorly because their density is greater than the vitreous. Oxane HD® (Bausch&Lomb, USA) is a mixture of SO (3,300 centistokes) with partially fluorinated olefin (1-perfluorooctyl-5-methylhex-2-ene)(RMN3), clear oil immiscible in water with a relative density of 1.02 g/cm^3^. Another HSO commercially available is Densiron 68 (Fluoron®, Geuder, Germany), a mixture of perfluorohexyloctane (F6H8) and 5,000 centistokes SO with a density of 1.06 g/cm [3, 8].

From our point of view, it is the proper vitreous tamponade for patients unable of face down positioning with high risk for PVR in inferior retinal detachments. Some examples are patients with cognitive impairment such as autism or dementia, obesity, spinal osteoarthritis, previously superior glaucoma drainage valve implant and inferior retinal detachment in patients with uveitis. It is important to mention that these complex cases require experienced surgeon able to identify and peel off all membranes and residual traction, especially anterior PVR, or associate scleral buckling or retinotomies/ retinectomies to relief longitudinal and tangential traction.

One of the concerns regarding HSO is about their removal. Because of its density, it progressively concentrates in the posterior pole during its removal and should be done under direct visualization and continuously aspirated inside the bubble during the whole procedure. In our experience, Oxane HD has higher viscosity and is more difficult to remove than Densiron. Intraoperative care is important to ensure there is no residual PFCL and avoid direct PFCL/HSO exchange to avoid sticky SO formation. In this series, there was not any retinal tear or early hypothony during HSO removal.

### Clinical postoperative outcomes

The most common complication after HSO surgeries is development of cataract [9]. For this reason, all phakic patients underwent combined phacoemulsification during the first procedure using HSO. We also believe this step facilitates access to vitreous base shaving which is essential for removing all the anterior vitreous. Another complication is the development of epiretinal membrane proliferation. Some authors believe it may be prevented if endotamponade is early removed [10]. Moreover, some authors report intraretinal and subretinal fibrosis from 10 to 29.2% in complex retinal detachment cases treated with HSO. These conditions are sight-threatening and mentioned to occur in patients with previously PVR [11–12]. In our cohort, two (6.9%) patients developed epiretinal membrane and intra/subretinal fibrosis were not detected.

Another described complication of HSO is inflammation and oil emulsification in the anterior chamber, especially in scenarios where the blood-retinal barrier is compromised or when SO is used over the long term [10, 13–14]. Recent metanalysis did not identified increased risk for inflammation or oil emulsification with HSO comparing with standard SO [15]. There are several theories about the mechanisms for development of intraocular inflammation: direct toxicity and immunogenicity (delayed type IV hypersensitivity), toxicity due to impurities or instability of the agent, oil emulsification, and mechanical injury due to gravity. [14, 16–17]. In our series, there was two patients which had retinal detachment with bad PVR and developed early emulsification before 3 months. These cases were treated with HSO removal or exchange for standard SO. Sixteen patients had Oxane HD for more than six months because social/health issues or unwilling to attend to further surgery due to bad visual recovery. Four of them developed later emulsification.

IOP elevation is another common postoperative complication. Previous studies have reported no difference in IOP elevation when comparing standard 5,000 cSt SO and HSO surgeries [18]. In the current series, IOP rise was frequent while the patients had the HSO and were well controlled with hypotensive eyedrops. Two patients needed glaucoma procedures after receiving dexamethasone implants for macular edema several months after SO/HSO removal and one patient needed cyclophotocoagulation for uncontrolled neovascular glaucoma four years after Oxane removal and replacement for standard SO. At the last follow up, over than a third of the patients (38.7%) were kept with hypotensive eye drops.

Furthermore, aphakic patients require closer follow-ups due to the risk of corneal decompensation. There were two aphakic patients in our study: one developed bullous keratopathy and the other developed globe atrophy. There was another patient that required corneal graft transplantation which had previously undergone glaucoma valve implant and needed two cyclophotocoagulation procedures.

The last but not the least, unexplained visual loss has been described associated in patients with SO/HSO more often than those with expandable gases. It is controversial if the incidence of unexplained visual loss after HSO removal has the same or different incidence with standard SO [19, 20]. Some investigators have theorized a sudden change in potassium ion concentrations, direct phototoxicity from the operating microscope, or the refractive effect of a shrinking SO bubble as possible pathogenesis. Particularly in HSO removal, the bubble shrinkage is concentrated near the posterior pole opposite to the position of the standard SO near the lens, which apparently may not be the cause. In our study, there was not any patient with unexplained visual loss, but it is important to highlight this pathology could be misdiagnosed because the cohort included patients with bad visual prognosis and recurrent retinal detachments.

Recent studies by Moussa et al. reported that the incidence rates of cataract, macular epiretinal membranes, IOP elevation, inflammatory reactions, and emulsification of HSO and standard SO were similar. On the other hand, some of these of these complications are more common in severe cases when there is need of retention of the SO. Moreover, it was noted less retinectomy rates in favor of HSO when compared to standard SO [14, 20].

In several cases, the HSO remained in the eye for longer than 6 months without substantial complications. This is longer than reported in other studies (usually 3–6 months) because most patients had complex retinal detachments or persistent postoperative hypotony. Some of these patients had preserved ambulatory vision (≥ 20/800) and were afraid of consequent surgeries [2, 10]. In our series, there were 11 patients unwilling or incapable to be submitted to SO or HSO removal surgery and their tamponade for over a year, especially due to bad visual prognosis such as age macular degeneration, optic nerve atrophy, or bullous keratopathy. Only one of these eleven patients required further surgery: a corneal transplant after multiple glaucoma surgeries.

Regarding anatomic success and visual outcomes, HSO has been reported to be neither inferior nor superior compared with standard SO. It is important to highlight that HSO cannot prevent PVR formation, although it shifts the PVR formation to the superior retina. Future studies about drugs to inhibit PVR formation are needed as alternative/adjuvant therapies for these severe cases [3, 5, 21, 22]. In the current series, poor VA outcome at the final follow-up visit was associated with PVR formation and multiple surgeries, a finding that agreed with previously reported data [9].

This study has some limitations. It is retrospective and selected patients from 2014 onward from one single center. This series included some patients with bad visual prognosis that explains the re-detachments rate and the reason some patients were left with HSO for over than 6 months period. All patients at this study were treated with Oxane HD HSO and the results should not be addressed for other HSO such as Densiron. Furthermore, careful analysis of retinal layers on OCT should be evaluated in future prospective studies.

We concluded that HSO is safe and useful for complex retinal detachment patients with inferior tears and PVR. Follow up of these patients is advised postoperatively due to the ocular complications, particularly elevated intraocular pressure and emulsification.

## Data Availability

Data is available upon reasonable request.

## Abbreviations

SO: silicone oil
PVR: proliferative vitreoretinopathy
HSO: heavy silicone oil
PFCL: perfluorocarbon liquids
IOP: intraocular pressure
BCVA: best corrected visual acuity
logMAR: logarithm of minimum angle of resolution
OCT: optical coeherence tomography
